# Supplementary material for: Tannic Acid Improves Renal Function Recovery after Renal Warm Ischemia–Reperfusion in a Rat Model
Source: Biomolecules. 2020 Mar 12;10(3):439. doi: 10.3390/biom10030439 (PMC7175177; doi:10.3390/biom10030439)
Supplement: Supplementary file 1 [file biomolecules-10-00439-s001.pdf]

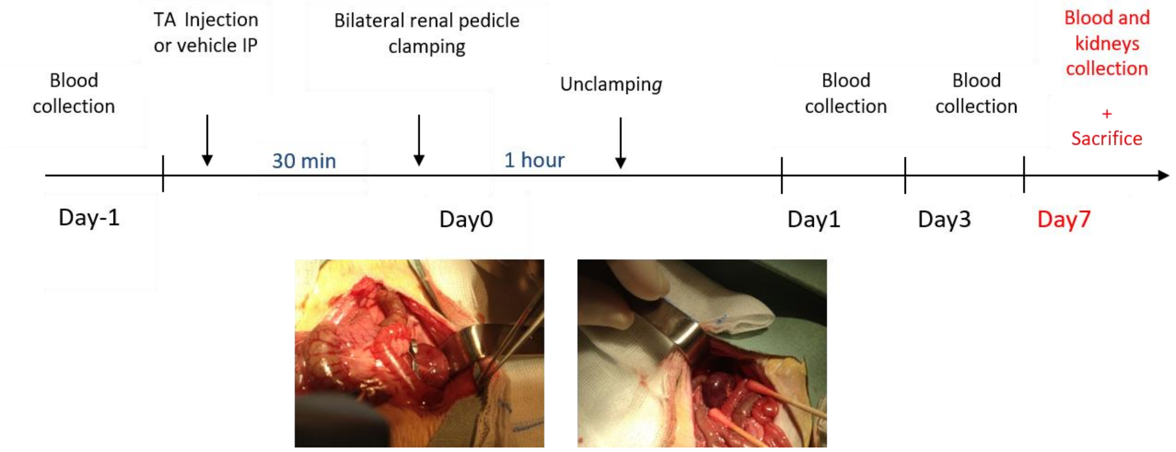

Supplementary Figure S1. Schematic representation of renal bilateral warm ischemia-reperfusion rat model

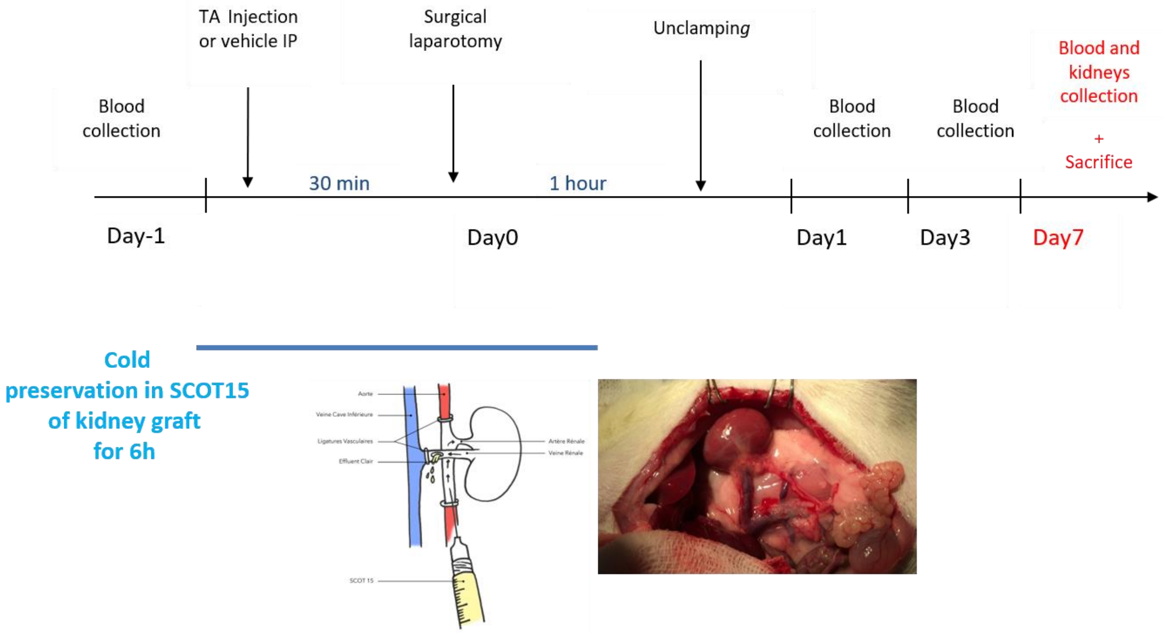

Supplementary Figure S2. Schematic representation of cold ischemia-reperfusion rat model: renal allotransplantation in recipient

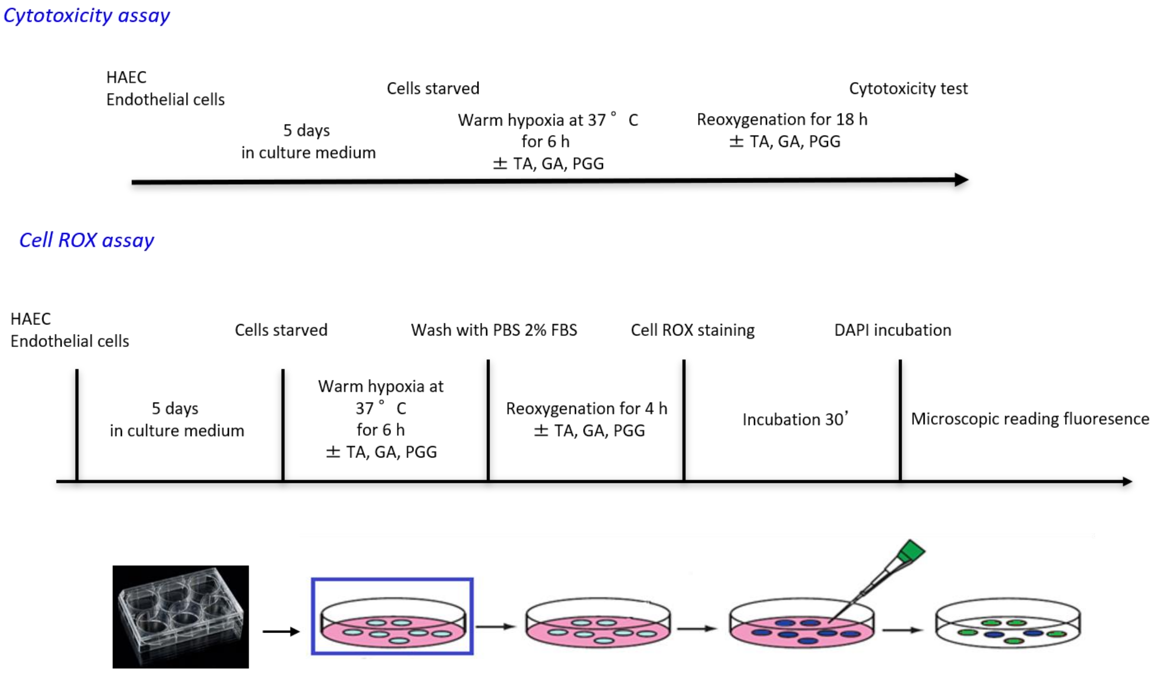

Supplementary Figure S3. Schematic representation of in vitro cytotoxicity and Cell Rox assays
